# Functional genetic diversity of domestic and wild American mink (*Neovison vison*)

**DOI:** 10.1111/eva.13061

**Published:** 2020-07-30

**Authors:** Kimberley Y. Morris, Jeff Bowman, Albrecht Schulte‐Hostedde, Paul J. Wilson

**Affiliations:** ^1^ Environmental and Life Sciences Graduate Program Trent University Peterborough ON Canada; ^2^ Wildlife Research and Monitoring Section Ontario Ministry of Natural Resources and Forestry Peterborough ON Canada; ^3^ Department of Biology Laurentian University Sudbury ON Canada; ^4^ Department of Biology Trent University Peterborough ON Canada

**Keywords:** domestication, functional gene, introgression, mink, mustela, *Neovison vison*

## Abstract

The release of domestic organisms to the wild threatens biodiversity because the introduction of domestic genes through interbreeding can negatively impact wild conspecifics via outbreeding depression. In North America, farmed American mink (*Neovison vison*) frequently escape captivity, yet the impact of these events on functional genetic diversity of wild mink populations is unclear. We characterized domestic and wild mink in Ontario at 17 trinucleotide microsatellites located in functional genes thought to be associated with traits affected by domestication. We found low functional genetic diversity in both mink types, as only four of 17 genes were variable, yet allele frequencies varied widely between captive and wild populations. To determine whether allele frequencies of wild populations were affected by geographic location, we performed redundancy analysis and spatial analysis of principal components on three polymorphic loci (AR, ATN1 and IGF‐1). We found evidence to suggest domestic release events are affecting the functional genetic diversity of wild mink, as sPCA showed clear distinctions between wild individuals near mink farms and those located in areas without mink farms. This is further substantiated through RDA, where spatial location was associated with genetic variation of AR, ATN1 and IGF1.

## 1 INTRODUCTION

Domesticated organisms can interbreed with the closely related wild species they derived from, which threatens biodiversity by reducing the local adaptation of the wild species, leading to outbreeding depression. For example, in Scandinavia, interbreeding between fur‐farm fox (*Vulpes lagopus*) and native arctic fox can potentially disrupt the timing and number of kit births, a trait otherwise well adapted to the fluctuation in food availability (Norén, Dalén, Kvaløy, & Angerbjörn, 2005; Tannerfeldt & Angerbjörn, 1998). Similarly, interbreeding between wildcat (*Felis silvestris*) and domestic house cat (*Felis catus*) produces hybrids that are more generalized foragers than the wildcat, leaving the wildcat at a disadvantage in a habitat experiencing fluctuations in prey (Oliveira, Godinho, Randi, Ferrand, & Alves, 2008; Rhymer & Simberloff, 1996). Hybridization of wild and domestic furbearers in Europe has been documented between fur‐farm arctic fox and wild arctic fox, fur‐farm red fox (*Vulpes vulpes*) and endemic red fox, and domestic ferret (*Mustela furo*) and polecats (*Mustela putorius*; Costa et al., 2013; Norén et al., 2005; Sacks, Moore, Statham, & Wittmers, 2011). In North America, domestic American mink (*Neovison vison*) have been continually released or escape from captivity and are introgressing into wild mink populations (Kidd, Bowman, Lesbarrères, & Schulte‐Hostedde, 2009).

In Canada, domestication of American mink (*Neovison vison*) began in 1866 as the fur farming industry expanded from its origins in Ontario. In recent years, approximately 2.2–2.6 million mink pelts are produced each pelting season, contributing almost $200 million annually to the Canadian economy (Statistics Canada, 2010). With such high economic value, it is important to both mink farmers and consumers that mink pelts are of the highest quality, with fur that is thick, soft and glossy (Obbard, 1987). As a result of over 150 years of intentional breeding for these traits, captive American mink are phenotypically and genetically different than their free‐ranging counterparts: farmed mink produce thicker pelts, are less aggressive and are approximately twice the size of free‐ranging mink (Belliveau, Farid, O’Connell, & Wright, 1999; Malmkvist & Hansen, 2002). Particularly easy to distinguish is pelt colour: wild mink are usually dark brown in colour, whereas farmed mink have a wide range of whites, browns, blacks and spotted pelts (American Fur Breeder, 1959; Joergensen, 1985). Artificial selection and other genetic mechanisms experienced in captivity differ from those in a natural environment, which may result in animals with lowered fitness when introduced to the wild (Hansen, 2002; Lynch & O’Hely, 2001; Price, 1984). If large numbers of domestic individuals repeatedly escape and interbreed with native populations, as reported in Ontario with domestic mink, this could result in the introgression of maladaptive alleles into the wild which may lower the fitness of the native population (Bowman, Kidd, Gorman, & Schulte‐Hostedde, 2007; Hansen, 2002; Hindar, Ryman, & Utter, 1991).

To assess whether functional gene complexes of domestic mink are compromising those of the introgressant mink populations, we first sought to characterize functional genetic diversity in wild and domestic American mink using microsatellites found in coding regions of the genome and in promoter regions that are noncoding but may be linked to functionally important genes. Sequence polymorphisms in coding regions are responsible for the variation in traits exhibited among individuals—variation that can be targeted by artificial selection and other genetic mechanisms (Borštnik & Pumpernik, 2002). Areas under artificial selection are especially interesting as we expect to see the greatest genetic difference between wild and domestic populations at these loci. With samples from multiple farm and wild sites across Ontario, our first objective was to determine whether trinucleotide microsatellite markers of identical repeat size differed in sequence. Slip‐strand mispairing (SSM), a mutation process that results in the addition or deletion of a tandem repeat unit, occurs both in the farm and in the wild (Fan & Chu, 2007; Levinson & Gutman, 1987). SSM is known to cause frameshift mutations, which can change the gene function and thus impact survival in the wild (Fan & Chu, 2007). However, because selection against new mutations causing a change in amino acid repeat sequence or length may be less constrained in captivity, signatures of SSM might be more prevalent in domestic mink. Therefore, we expect to see sequence differences in alleles of the same size between domestic and wild mink.

Secondly, we sought to assess how domestication has affected functional genetic variability in American mink by genotyping individuals at loci thought to be targeted by artificial selection. Studies have shown that domestication can lower genetic diversity in neutral loci within breeding lines; however, little is known about how it affects the genetic diversity of functional genes (Belliveau et al., 1999). Domestication is driven by intense artificial selection which can increase the frequency of an allele until it is fixed in the population, and given American mink undergo intense artificial selection (e.g. for large size), we are likely to see a small range of alleles associated with these traits. In addition to artificial selection, the main genetic mechanisms influencing the domestication of mink are as follows: natural selection, relaxation of natural selection, inbreeding and genetic drift. The effects of domestication on genetic diversity vary depending on the selection pressure placed on different traits. For example, natural and artificial selection, inbreeding and genetic drift are thought to reduce genetic variability, whereas relaxed natural selection is thought to increase genetic variability through the production of new alleles (Price, 1984). Therefore, the amount of functional genetic variability in farmed mink relative to wild mink should differ by trait and the conditions under which that trait is maintained on each individual farm.

Our third objective was to determine how functional genetic diversity of wild mink populations is affected when domestic mink are introduced. Captive American mink are continuously being released and escaping to the wild (Joergensen, 1985). Bowman et al. (2007) estimated that 38% of free‐ranging mink harvested per province per year in Canada were of domestic origin. Furthermore, domestic escapees are persisting within wild mink populations, with one sampled wild population containing only 22% wild individuals (Kidd et al., 2009). Backcrossing of domestic–wild hybrid mink occurs in both directions, confirming that domestic alleles at neutral loci are being introgressed into wild mink populations (Kidd et al., 2009). We hypothesized that the phenotypes unique to captive mink are maladaptive to life in the wild; therefore, alleles common in captive mink are selected against in the wild and consequently occur in wild mink populations at relatively low frequencies. We consider that alleles common in domestic mink confer low fitness in wild mink and therefore should only occur in close proximity to mink farms as a result of recent introgression. We quantified this by testing genetic variation against mink farm density per township using redundancy analysis and spatial analysis of principal components.

## 2 MATERIALS AND METHODS

### 2.1 Microsatellite genotyping

**Figure 1:**
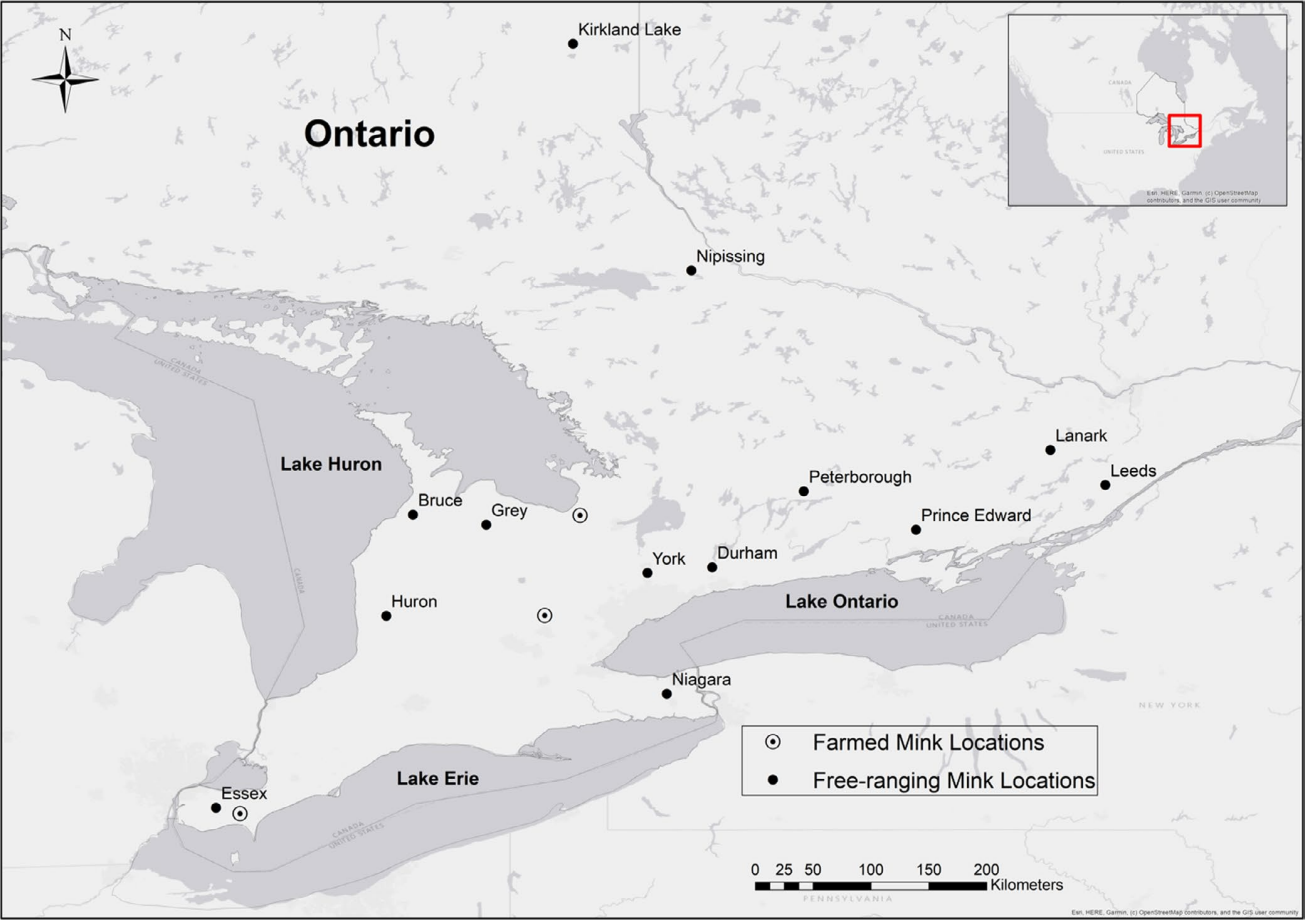
American mink samples were collected from 13 sites in Ontario from 2006 to 2015. Sites ranged from Kirkland Lake to Point Pelee Provincial Park. Black symbols represent the centroid of the area where free‐ranging mink were sampled, and white symbols represent the centroid of townships where we sampled mink farms. The majority of mink farms in Ontario occur within 200 km of Lake Erie

Free‐ranging American mink were sampled in locations across Ontario during 2005–2015. The 144 samples originated from Bruce County, Durham, Essex County, Grey County, Huron County, Kirkland Lake, Leeds, Niagara, Nipissing, Perth, Peterborough County, Wellington County and York (Table 1; Figure 1). Captive mink (*n* = 143) were donated by three farms in Ontario and a pelting service in Nova Scotia. Samples were separated into five groups: Nova Scotia domestic black (NSB), Ontario domestic black (ONB), Ontario domestic brown (OND), wild (Wild) and free‐ranging Ontario mink of mixed origin that cannot be classified as either fully domestic or fully wild (Hybrid) based on Bayesian assignment tests carried out in a previous study (Kidd et al., 2009). Individuals with a mean membership coefficient *q* ≥ 0.8 were assigned as wild, those with *q* ≤ 0.2 were domestic, and the remaining (0.2 > *q* < 0.8) were considered hybrid though they may have been backcrossed to domestic or wild groups (Kidd et al., 2009). DNA was extracted from spleen and liver using a DNeasy Blood & Tissue Kit (Qiagen) and quantified using a Quant‐iT Picogreen^®^ dsDNA Assay Kit (Invitrogen™) on the BMG FLUOstar microplate‐reader system (BMG‐LabTech).

**Table 1:** Number of free‐ranging or captive American mink (*Neovison vison*) sampled per colour line in farms or per location

Type	County	Individuals sampled
Free‐ranging	Bruce	5
	Durham	5
	Essex	6
	Grey	26
	Huron	6
	Lanark	5
	Leeds	5
	Kirkland Lake	47
	Niagara	7
	Nipissing	1
	Peterborough	18
	Prince Edward	4
	York	9
Domestic	Nova Scotia (Black)	76
	Ontario (Black)	56
	Ontario (Brown)	11

Genes were selected for their putative adaptive potential. Genes related to functions such as behaviour, development and stress were targeted because they can be artificially selected for by mink ranchers and consequently may show variation between captive and wild mink. We downloaded DNA sequences from the National Center for Biotechnology Information (NCBI) and designed primers (*n* = 38) around regions containing 6 + repeat units using Geneious 9.0.5 (Kearse et al., 2012). Primers were manufactured by Integrated DNA Technologies. Primers were tested in a PCR cocktail consisting 1 mM PCR buffer (10×), 2 mM MgCl_2_, 0.2 mM dNTPs, 0.1 mg/ml BSA, 0.2 µM each forward and reverse primer, 0.04 U Taq polymerase and 5 ng DNA for a total reaction volume of 8 µl/sample. Thermal‐cycling conditions consisted of the following: 95°C for 10 min, 94°C for 30 s, 55°C for 1 min and 72°C for 1 min; 30 cycles of 94°C for 30 s, 50–65°C for 1 min and 72°C for 1 min, followed by 65°C for 15 min. Products were visualized on a QIAxcel capillary electrophoresis system (Qiagen) using a DNA Screening Kit. Primers that successfully amplified their target (*n* = 17, Table A1 in Appendix) were then tested for polymorphisms using a subset of samples that was comprised of wild and multiple lines of captive American mink, both males and females, in order to represent the complete sample set and encompass multiple levels of variation (between sex, colour and origin [domestic vs. wild]). The same PCR cocktail was prepared, this time containing a fluorescently labelled forward primer, and amplified with an annealing temperature of 55–61°C depending on the primer (Table 2). New primers were fluorescently labelled with either HEX or 6‐FAM (Integrated DNA Technologies, Inc.) or NED (Applied Biosystems). Genotyping products were suspended in HiDi formamide and the GeneScan size standard LIZ500 (Applied Biosystems) and visualized on an ABI3730 DNA Analyzer (Applied Biosystems). Genotypes were scored using GeneMarker v1.70 (SoftGenetics) software. I). Of these 17 loci, only four displayed length polymorphisms: androgen receptor (AR), atrophin 1 (ATN1), insulin‐like growth factor 1 (IGF1) and transducer of ERBB2 (TOB1). The remaining 13 loci did not show variation within or among mink types.

**Table 2:** Location, annealing temperature and observed alleles of four functional microsatellite loci (AR, ATN1, IGF‐1 and TOB1) in wild, hybrid and domestic mink from three lines: Nova Scotia black (NSB), Ontario black (ONB) and Ontario brown (OND)

Locus	Location	*T*_a_ (°C)	Observed alleles (bp)				
			NSB	ONB	OND	Wild	Hybrid
ATN1	Exon	55	226, 232, 235	226, 235	232, 235	229, 232, 235	232, 235
AR	Exon	61	289, 295, 298, 301, 304	289, 298, 301, 304	289, 295, 298, 301	292, 295, 298, 301, 304	295, 298
IGF‐1	Promoter	54.2	103, 105, 107, 111	103, 105, 107	103, 105, 107	97, 99, 101, 103, 105, 109, 111	103
TOB1	Exon	61	242, 245	242, 245	242, 245	242, 245	242, 245

A total of 291 American mink were profiled at AR, ATN1, IGF1 and TOB1. Individuals (*n* = 8) that could be scored at fewer than 70% of the target loci were eliminated from further analysis. We tested for Hardy–Weinberg equilibrium (HWE) and linkage disequilibrium (LE) with GENEPOP 4.5.1 using Markov chain parameters of 5,000 iterations with 10 repetitions (Rousset, 2008). Effective number of alleles was determined using an allelic richness test in Fstat 2.9.3.2 (Goudet, 1995). Population‐specific variations in allele distributions were tested with a principal components analysis (PCA) in RStudio 3.3.0 using the adegenet package (Jombart, 2008). Also analysed were 15 neutral microsatellite loci previously used to assess population genetic structure and diversity in the same individuals (Tables A2 and A3 in Appendix; Beauclerc, Bowman, & Schulte‐ Hostedde, 2013).

### 2.2 Microsatellite sequencing

To test for sequence polymorphisms in same‐sized alleles, we sequenced androgen receptor (AR) and atrophin 1 (ATN1) alleles in 43 and 135 individuals, respectively. The AR alleles sequenced included: the following 292 (*n* = 4), 295 (*n* = 6), 298 (*n* = 23), 301 (*n* = 8) and 304 (*n* = 2). The ATN1 alleles sequenced were 229 (*n* = 1), 232 (*n* = 9) and 235 (*n* = 125). Amplified products were purified with ExoSap (New England Biolabs) and prepared for sequencing using a BigDye Terminator v3.1 Cycle Sequencing Kit (Applied Biosystems). Sequencing reaction products were purified with ethanol precipitation, then suspended in HiDi formamide and sequenced on an ABI3730 DNA Analyzer (Applied Biosystems). Sequence data were analysed with Molecular Evolutionary Genetics Analysis v6.06 (MEGA) software (Tamura et al., 2011).

### 2.3 Apportioning genetic variation to environmental variables

Redundancy analysis (RDA) was used to identify genetic variation significantly associated with environmental gradients. Using the *rda* function of the “vegan” package in RStudio 3.5.2, the wild and hybrid genetic data sets were used as the response matrix Y, and a set of anthropogenic and spatial variables (mink farm density, road density, latitude and longitude) was used as the explanatory matrix X (Oksanen et al., 2019). Mink farm density was used to determine whether free‐ranging mink found in areas that have a high density of mink farms exhibit different allele frequencies from mink in areas of low density of mink farms. Road density functioned as a proxy for human population density to test whether allele frequencies of American mink varied according to changes in human population levels. Since most mink farms in Ontario occurred in the south‐west of the province, we were interested in potential north–south or east–west gradients in alleles of domestic origin, and therefore, latitude and longitude were included as a measure of location. Latitude has previously shown to be a good predictor of a free‐ranging mink’s probability of being classified as domestic based on neutral markers (Beauclerc et al., 2013). To obtain mink farm density values, we used ArcMap 10.1 to determine the township each sample was associated with and then divided the number of mink farms in that township by the township area (km^2^; Statistics Canada, 2006). Similarly, road density values were obtained by dividing the sum of the lengths of all the roads (km) in the township by the township area (km^2^). RDA was completed for each locus on 159 individuals, and an additional RDA was performed on the same individuals using the entire neutral data set. Outlier alleles were identified using the test statistic Mahalanobis Distance *D*, which was computed using the *covRob* function of the “robust” package in R (Capblancq, Luu, Blum, & Bazin, 2018; Wang et al., 2017). Then, we performed a second RDA with only outlier alleles (*q*‐values < 0.1) to identify the environmental variables that are most correlated with the genetic variation of those alleles. Next, to determine whether allele frequencies varied by location, we evaluated the principal components of each functional locus and neutral data set over geographic space using spatial analysis of principal components (sPCA) using the “adegenet” package (Jombart, 2008).

## 3 RESULTS

### 3.1 Microsatellite genotyping

Of the 38 primer pairs tested, 17 amplified a single amplicon at the size expected with no evidence of nonspecific amplification or smearing (Tables A1 and A4 in Appendix). Of these 17 loci, only four displayed length polymorphisms: androgen receptor (AR), atrophin 1 (ATN1), insulin‐like growth factor 1 (IGF1) and transducer of ERBB2 (TOB1). A total of 287 American mink were profiled at AR, ATN1, IGF1 and TOB1 resulting in 19 alleles and a mean of 4 alleles per locus (range 2–8; Tables 2 and 3).

**Table 3:** Allele frequencies of four functional microsatellite loci (AR, ATN1, IGF‐1 and and TOB1) in wild, hybrid and domestic mink from three lines: Nova Scotia black (NSB), Ontario black (ONB) and Ontario brown (OND)

Locus	Allele	Allele frequency				
		NSB	ONB	OND	Wild	Hybrid
AR (females)	289	0.050	0.020	0.250	0.000	0.000
	292	0.000	0.000	0.000	0.109	0.000
	295	0.020	0.000	0.125	0.182	0.333
	298*	0.830	0.900	0.500	0.427	0.666
	301	0.090	0.060	0.125	0.155	0.000
	304	0.010	0.020	0.000	0.127	0.000
Number of Individuals		55	25	4	56	3
ATN1	226	0.007	0.018	0.000	0.000	0.000
	229	0.000	0.000	0.000	0.094	0.000
	232	0.056	0.000	0.273	0.231	0.125
	235*	0.938	0.982	0.727	0.675	0.875
IGF‐1	97	0.000	0.000	0.000	0.035	0.000
	99	0.000	0.000	0.000	0.003	0.000
	101	0.000	0.000	0.000	0.024	1.000
	103*	0.899	0.911	0.909	0.792	0.000
	105	0.041	0.063	0.045	0.052	0.000
	107	0.041	0.027	0.045	0.021	0.000
	109	0.000	0.000	0.000	0.003	0.000
	111	0.020	0.000	0.000	0.069	0.000
TOB1	242	0.323	0.500	0.136	0.191	0.125
	245*	0.677	0.500	0.864	0.809	0.875
Number of Individuals		76	56	11	144	4

(*) Indicate significant (*p* < .0026) difference among populations.

**Figure 2:**
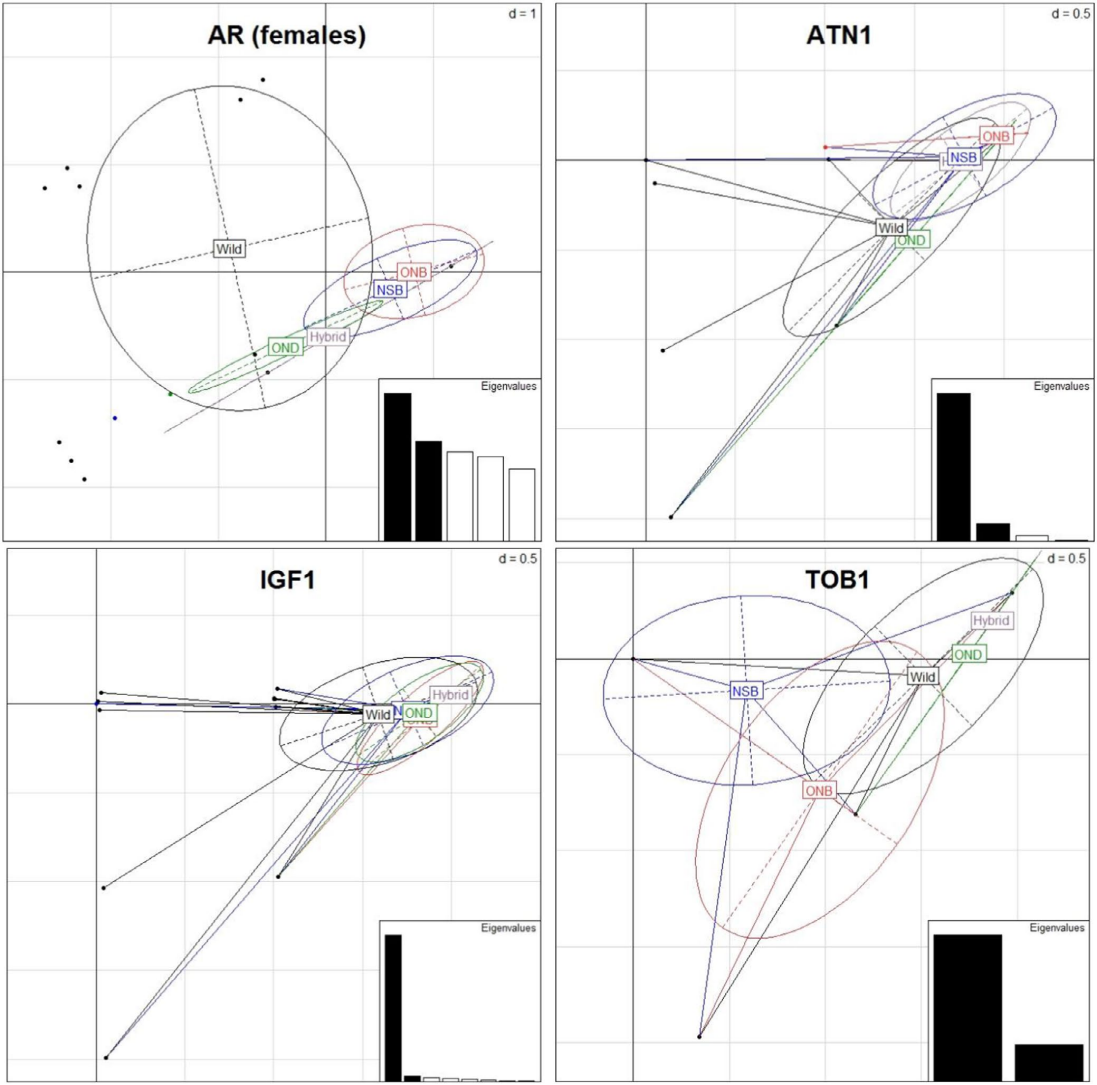
Biplots of PC1 versus PC2 for four functional genes of American mink: AR (females only), ATN1, IGF1 and TOB1 allele frequencies by population (Nova Scotia black (NSB), Ontario black (ONB), Ontario brown (OND), Hybrid and Wild). The majority of ellipses overlap, indicating a high degree of genetic similarity among populations. Eigenvalues represent the amount of variance retained by each principal component

We profiled the AR trinucleotide repeat in 135 females and 145 males and found six alleles (range 289–304). Population structure was assessed at AR using female mink only, as this gene is located on the X chromosome. Allele 289 was found only in domestic female mink and allele 292 only in wild mink. The frequency of allele 298 was significantly different between populations following Bonferroni correction, and only 43% of wild mink had allele 298, whereas it appeared to be approaching fixation in domesticated black mink (Table 3). The allele frequency disproportion between populations was supported by PCA, where only 25% of the domestic Ontario black mink ellipse overlapped with wild mink (Figure 2; Table A5 in Appendix). The wild mink population deviated from Hardy–Weinberg equilibrium following Bonferroni correction (Table 4).

**Table 4:** Number of alleles (Na), allelic richness (Ar), expected heterozygosity (He) and observed heterozygosity (Ho) for functional microsatellite loci in wild and domestic mink from three lines: Nova Scotia black (NSB), Ontario black (ONB) and Ontario brown (OND)

Locus	*F*_ST_		NSB	ONB	OND	Wild
AR (females)	0.121	Na	5	4	4	5
		Ar ± SD	1.63 ± 0.26	1.39 ± 0.35	2.77 ± 0.33	2.75 ± 0.53
		He ± SD	0.30 ± 0.05	0.19 ± 0.04	0.66 ± 0.08	0.73 ± 0.05
		Ho ± SD	0.32 ± 0.05	0.20 ± 0.05	0.50 ± 0.13	0.58 ± 0.04
Number of individuals			50	25	4	55
ATN1	0.111	Na	3	2	2	3
		Ar ± SD	1.73 ± 0.26	1.27 ± 0.35	2.00 ± 0.33	2.79 ± 0.53
		He ± SD	0.12 ± 0.06	0.04	0.40	0.48 ± 0.14
		Ho ± SD	0.10 ± 0.05	0.04	0.36	0.47 ± 0.13
IGF‐1	0.017	Na	4	3	3	8
		Ar ± SD	2.30 ± 0.26	2.04 ± 0.35	2.46 ± 0.33	3.46 ± 0.53
		He ± SD	0.19 ± 0.04	0.17 ± 0.06	0.17 ± 0.05	0.36 ± 0.03
		Ho ± SD	0.18 ± 0.04	0.18 ± 0.06	0.18 ± 0.05	0.35 ± 0.03
TOB1	0.096	Na	2	2	2	2
		Ar ± SD	2.00 ± 0.26	2.00 ± 0.35	1.99 ± 0.33	1.97 ± 0.53
		He	0.48	0.50	0.24	0.31
		Ho	0.45	0.49	0.27	0.28
Number of individuals			76	56	11	144

Profiling the ATN1 trinucleotide repeat in 282 individuals revealed four alleles in total (range 226–235). Adjusting for sample size, wild mink displayed the highest number of alleles (2.79), followed by OND (2.00) and NSB (1.73) and then ONB (1.27). Most alleles were shared between both wild and domestic American mink, apart from allele 226 found only in the domesticated black population and allele 229 found only in the wild population. Allele 226 was found at low frequencies (NSB 0.7%, ONB 1.8%) and may have gone undetected in other mink types. Allele 229 was also found at a low frequency (0.094%) in wild mink and therefore also may have gone undetected in domestic mink. Allele 235 displayed significant frequency difference following Bonferroni correction. We found no deviations from Hardy–Weinberg equilibrium after Bonferroni correction. ATN1 displayed significant linkage disequilibrium with the neutral locus Mvi072 in domesticated black mink in Nova Scotia; however, these two loci are not linked in any other population nor is ATN1 linked with any other locus (Table A6 in Appendix).

We characterized the IGF‐1 dinucleotide repeat in 285 individuals and found eight alleles (range 97–111). Wild mink had the highest number of alleles (3.46), followed by OND (2.46), then NSB (2.30) and ONB (2.04) after correcting for sample size. Alleles 97, 99, 101 and 109 were found only in wild American mink, granted at very low frequencies (<0.035) and therefore may have gone undetected in domestic mink. The frequency of allele 103 was significantly different between populations, yet principal components of IGF‐1 revealed high similarity of allele frequencies among domestic and wild populations (91%–94% ellipse overlap, Table A5 in Appendix). We found no evidence of linkage disequilibrium or HWE at Bonferroni‐corrected significance thresholds for each population.

We profiled the TOB1 trinucleotide repeat in 220 individuals and found two alleles (242 and 245). The frequency of allele 245 was significantly different between populations at Bonferroni‐corrected significance thresholds. Allele 245 was more commonly found than 242 in all mink types except for domesticated black mink in Ontario, where both alleles were found to occur in equal frequency (Table 3). Principal components of TOB1 suggest moderate allele frequency similarity between all populations except for between OND and wild mink (Figure 2; Table A5 in Appendix). We found no evidence for deviation from LE nor from HWE at Bonferroni‐corrected significance thresholds for each population.

American mink (*n* = 280) were profiled at 10–15 polymorphic neutral markers, which produced a total of 163 alleles and a mean of 11 alleles per locus (range 5–20; Tables A2 and A3 in Appendix). We found minimal evidence for deviation from Hardy–Weinberg equilibrium after Bonferroni correction: Mvi1302 deviated from HWE in domesticated black mink in Ontario, Mvi1321 and Mvi2243 deviated from HWE in domesticated brown mink in Ontario, and Mvi3102, Mvi099, Mvi2243, Mvi075, Mvi072 and Mvi1342 deviated from HWE expectations in wild mink. Loci seemed to be in linkage equilibrium in domesticated brown mink in Ontario; however, several pairs of neutral loci are in linkage disequilibrium in the remaining populations (Table A6 in Appendix).

### 3.2 Microsatellite sequencing analysis

Androgen receptor consisted of three repeat sections: a perfect repeat (“GCA”), followed by an imperfect repeat (“GGA GAC CAG TTC TCG”) and a second perfect repeat (“GCA”). Allele size variation was due to the number of repeats present in the first perfect (“GCA”) repeat section. For atrophin 1, a compound microsatellite containing (“CAG”) as well as (“CAA”) repeats, the size difference amongst alleles was due to the number of (“CAG”) repeats present. No nonsynonymous sequence differences between alleles of the same size were discovered.

### 3.3 Apportioning genetic variation to environmental variables

**Figure 3:**
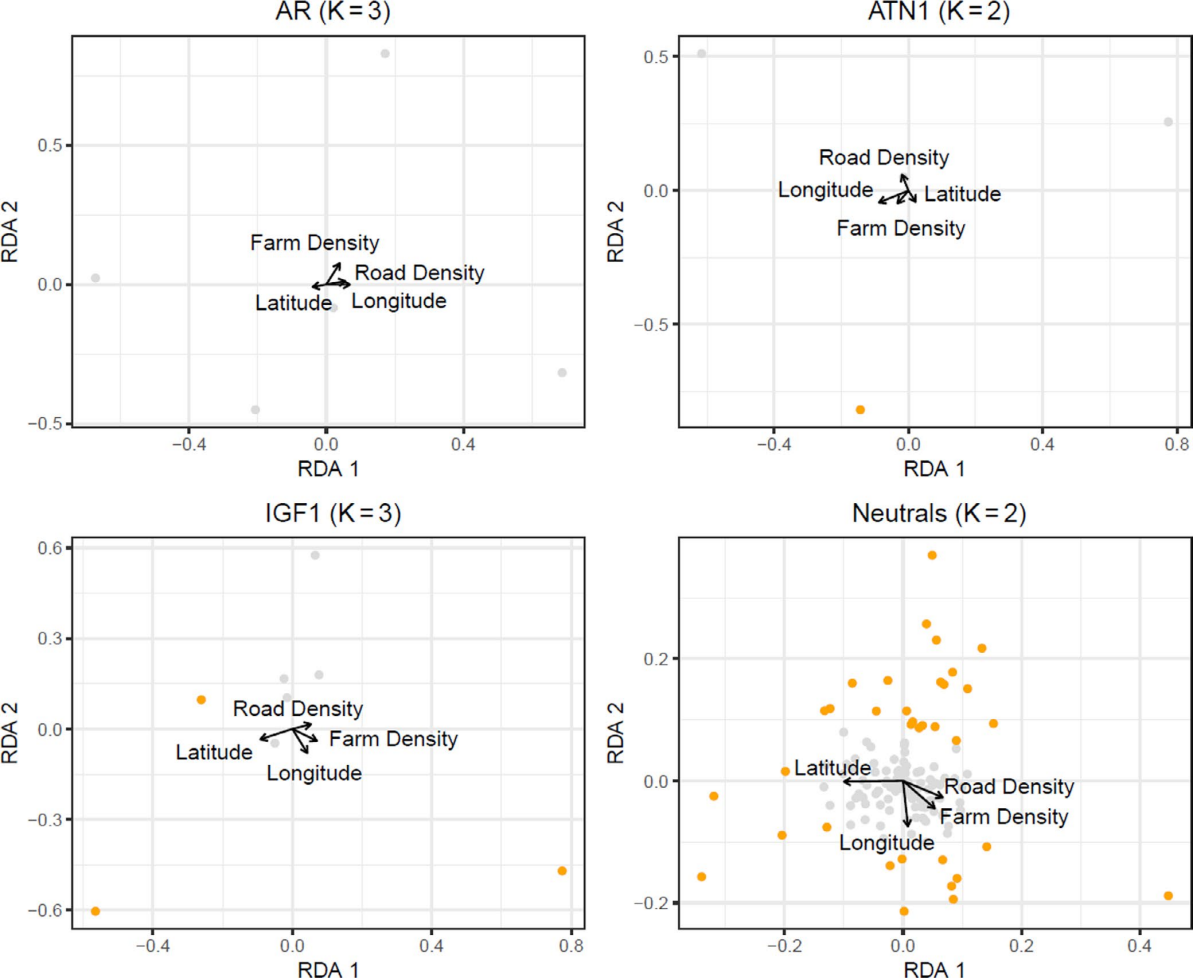
Redundancy analysis (RDA) of the relationship between American mink (Neovison vison) alleles and environmental variables at three functional genes (AR (females only), ATN1, IGF1) and a data set of neutral microsatellites. Alleles are represented by points, with outlier alleles coloured orange. Arrows indicate the direction and magnitude of the environmental variables

**Figure 4:**
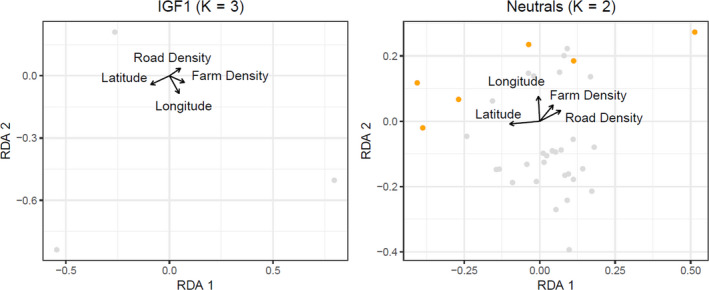
Redundancy analysis (RDA) of the relationship between American mink (Neovison vison) outlier alleles (identified in Figure 3) and environmental variables at IGF‐1 and a data set of neutral microsatellites. Alleles are represented by points, with outlier alleles coloured orange. Arrows indicate the direction and magnitude of the environmental variables

**Figure 5:**
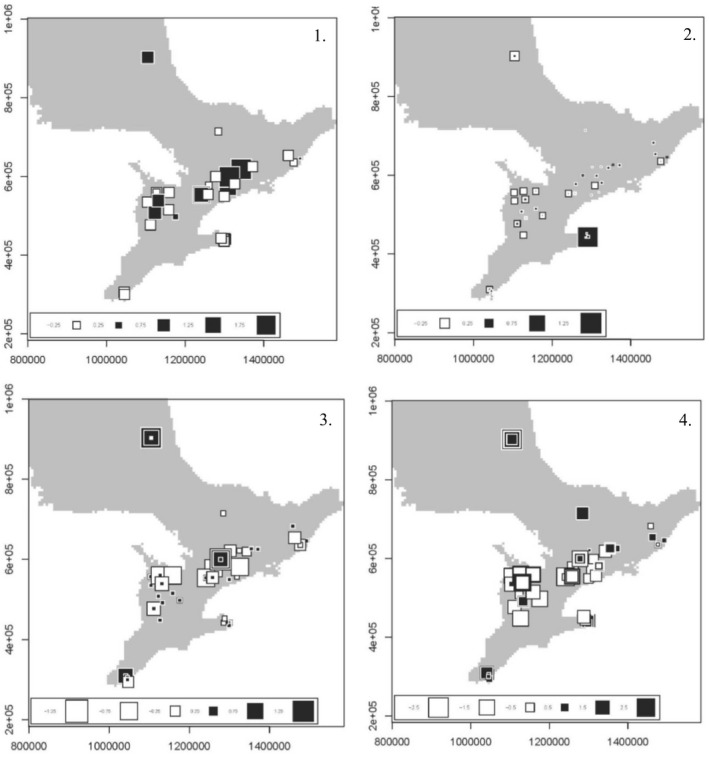
Spatial analysis of principal components of (1) AR (females only), (2) ATN1, (3) IGF‐ 1 and (4) neutral loci, performed on free‐ranging American mink (Neovison vison) in Ontario. Each square represents an individual mink. Genetic differentiation is steepest between individuals with large white squares and those with large black squares

Only a small proportion of genetic variation in each functional locus was affected by the environmental variables we tested (AR $R_{\text{adj}}^{2}$ = 0.0235, ATN1 $R_{\text{adj}}^{2}$ = 0.0114 and IGF1 $R_{\text{adj}}^{2}$ = 0.0271; Table 5). Longitude was a significant influence on genetic variation in AR (*R* ^2^ = .0413) and ATN1 (*R* ^2^ = .0211). With IGF1, it appeared that latitude was correlated with RDA1 (Figure 3). The follow‐up RDA performed on the three outlier alleles detected in IGF1 (103, 105 and 111) revealed that latitude did have an impact on genetic variation in IGF1 (*R* ^2^ = .0157), which was especially evident in RDA1, the axis that held most of the genetic variance (70%; Figure 4). The environmental variables had a small but significant effect on neutral loci ($R_{\text{adj}}^{2}$ = .0411); here, latitude, longitude and road density all explained significant genetic variation (*R* ^2^ = .0205; Table 5). We performed RDA on 35 outlier alleles detected in the neutral data set and found that most of the variance was explained by the first two axes (51% and 30%, respectively). Both axes were highly correlated with spatial location: RDA1 was correlated with latitude and RDA2 with longitude (Figure 4). Spatial analysis of principal components on neutral loci demonstrated a latitudinal gradient in genetic variation between individuals in northern and southern Ontario. The same pattern was observed in functional loci, though the latitude at which the north–south transition occurred varied by locus (Figure 5).

**Table 5:** Proportion of genetic variance of four functional microsatellite loci (AR, ATN1, IGF‐1 and TOB1) and a neutral data set explained by environmental variables (Farm Density, Road Density, Latitude and Longitude) using redundancy analysis on free‐ranging mink in Ontario

Locus	Full $\left(R_{\text{adj}}^{2}\right)$	Farm density (*R* ^2^)	Road density (*R* ^2^)	Latitude (*R* ^2^)	Longitude (*R* ^2^)
AR (females)	.0235	.0282	.0183	.0074	.0413*
ATN1	.0116	.0084	.0045	.0026	.0211*
IGF‐1	.0271	.0019	.0050	.0170	.0088
TOB1	.0297	.0083	.0425*	.0214	.0027
Neutrals	.0411*	.0066	.0135*	.0205*	.0014*
IGF‐1 (Outliers)	.0324	.0007	.0035	.0157*	.0095
Neutrals (Outliers)	.0669	.0052	.0185*	.02881*	.0158*

(*) Indicates variable is significant in explaining genetic variance.

## 4 DISCUSSION

Contrary to our prediction that slip‐strand mispairing causes sequence changes in domestic mink alleles, sequencing results from AR and ATN1 show no nonsynonymous change in repeat structure between alleles of the same size nor between alleles of varying size. However, it appears that domestication has affected the functional genetic diversity of captive mink populations. Captive mink displayed fewer alleles per locus than the wild population at AR, ATN1 and IGF1. In fact, allele frequencies were so different between captive and wild populations, that domestic release events affected functional genetic diversity of wild mink. As predicted, sPCA showed clear distinctions between wild individuals near mink farms and those located in areas without mink farms. This is further substantiated through RDA, where spatial location was associated with genetic variation of AR, ATN1 and IGF1.

### 4.1 Genetic diversity of wild and domestic American mink

Only four of 17 amplified loci displayed length polymorphisms: AR, ATN1, IGF1 and TOB; the remaining loci did not show variation within or between mink types. This low proportion of polymorphic loci is not unlike in some other mustelids. For example, a study on functional trinucleotide motifs in genes in fisher (*Pekania pennanti*) reported only 27% polymorphic loci (Greenhorn, 2016). American mink displayed a mean of four alleles per locus, whereas allelic diversity in fisher was three, showing an overall low variation in functional genes for both species. Furthermore, PCA revealed high similarity of functional allele frequency among mink populations at individual loci, regardless of which principal component was used, thus providing further evidence to the general trend of low variation in functional genes for American mink.

Androgen receptor (AR) encodes a protein involved in the regulation of androgen‐responsive genes (Bolton et al., 2007). AR is involved in male sexual development and has been linked to aggressive behaviour (Butovskaya et al., 2015; Hurd, Vaillancourt, & Dinsdale, 2010). We speculate the significant frequency difference in AR allele 298 between wild and domestic mink is due to selective breeding for lower aggression level. Multiple studies report that when breeding mink for domesticated behaviour, aggressive reaction to humans varied from generation to generation, but showed a general decline throughout the selection period (Kizhina et al., 2017; Kulikov et al., 2016). It is likely that in choosing less aggressive animals as the following years breeding stock, mink farmers in Ontario curated herds that are more docile than their wild counterparts. Further work genotyping AR in mink of known aggression level could help to determine whether allele 298 is associated with lower aggression levels.

Insulin‐like growth factor 1 (IGF‐1) encodes a hormone similar in structure to insulin and is involved stimulating cell growth and inhibiting cell death in almost every cell in the body (Davison, de Blacquière, Westley, & May, 2011; Murray, Zheng, Gu, & Xiao, 2003). Mutations of IGF‐1 can cause abnormalities in metabolism, stature and hearing (Aguirre, De Ita, De Garza, & Castilla‐Cortazar, 2016; Riguelme et al., 2010). Eight IGF‐1 alleles of lengths ranging from 97 to 111 were found in wild mink, whereas domestic mink in Ontario exhibited only the three mid‐sized IGF‐1 alleles (103, 105 and 107). The frequency of allele 103 was significantly different between wild and domestic mink; this shift towards allele 103 and other mid‐range IGF‐1 alleles may have been a result of artificial selection for large size, though this is purely speculative.

Atrophin 1 (ATN1) encodes a protein thought to be involved in kinase binding, toxin receptor binding and transcription corepressor activity (Wood et al., 2000). The exact function of ATN1 is unknown, but an expansion of the trinucleotide repeat within this gene is responsible for dentatorubral–pallidoluysian atrophy (DRPLA), a neurodegenerative disorder similar to Huntington’s disease (Wood et al., 1998). Transducer of ERBB2 (TOB1) encodes a protein thought to function as a tumour suppressor (Zhang et al., 2016). We cannot attribute allele frequency differences between populations at either ATN1 or TOB1 to a particular function.

Overall, captive American mink displayed a lower number of alleles, at both neutral and functional loci, than wild mink. In addition to artificial selection, this loss of variation in captive mink and the difference in allele frequencies between wild and captive mink may be a result of other genetic mechanisms influencing domestication, namely inbreeding, relaxed natural selection or genetic drift. Natural selection accompanies artificial selection in captive populations, usually in the form of reproductive failure or increased infant mortality rate due to inbreeding depression (American Fur Breeder, 1959; Belliveau et al., 1999; Demontis et al., 2011; Price, 1984). While inbreeding is usually a chance phenomenon, intentional breeding of related individuals is practiced in an attempt to obtain or maintain a particular characteristic (Belliveau et al., 1999; Price, 1984). For instance, many pelt colours, known as colour phases, are recessive to the standard brown and must be line bred; however, this breeding strategy can result in inbreeding at loci controlling fur quality traits as well as at traits linked with such loci (Belliveau et al., 1999; Gregorius, 1980).

American mink possess many traits that are thought to impede the domestication process: solitary and territorial social structure, altricial young, extreme wariness to man and extreme agility (Hale, 1969). These behavioural characteristics are not advantageous to life in captivity, so it is very likely that the first mink herds experienced intense natural selection in the first few generations following capture. However, many characteristics that were once heavily selected for or against in nature are done so at a much lower intensity in captivity. For example, captive mink do not compete for resources, avoid predation or compete for mates; these behaviours essential for survival in the wild lose their significance in captivity, and as a result, genetic and phenotypic variability for these traits are likely to increase (Price, 1984). Relaxed natural selection may result in an increased frequency of alleles that are normally selected against in nature, and while these deleterious alleles may have no effect in a farm environment, they could have negative impacts on mink survival in the wild (Lynch & O’Hely, 2001; Price, 1984; Rhymer & Simberloff, 1996; Snyder et al., 1996). Androgen receptor allele 289 was found only in captive mink populations, and while it is possible the trait associated with this allele is the target of artificial selection, the fact that it was found in each captive population regardless of breeding line suggests captivity has relaxed the natural selection normally acting on this trait in the wild. However, it is not known whether AR allele 289 originated in captivity or existed in the wild before mink were sampled for this study.

Genetic drift is the change in frequency of an allele in a population due to random sampling. The phenomenon is unpredictable and tends to reduce genetic variability within populations while increasing genetic variability between populations (Dobzhansky & Pavlovsky, 1957). Considering the colour‐phase strategy in which mink are bred, genetic drift may affect mink differently according to their line and farm. Drift is a common occurrence in captive groups of animals founded by small isolated populations; therefore, captive American mink are highly susceptible to this genetic mechanism (American Fur Breeder, 1959; Price, 1984).

### 4.2 Apportioning genetic variation to environmental variables

Traditionally, to evaluate genetic differentiation, conservation genetic studies use *F*_ST_, but this method requires defining populations, which is unrealistic to do for wild populations that show continuous genetic gradients (Lewontin & Krakauer, 1973; Martins, Caye, Luu, Blum, & François, 2016). To avoid this impracticality, we used RDA, an individual‐based multivariate method that can detect significant genetic differentiation through the identification of outlier alleles using the test statistic Mahalanobis distance. We then performed a secondary RDA using the outliers defined by Mahalanobis distance, effectively “creating a space in which we can identify the environmental variables that are most correlated with putatively adaptive variation” (Capblancq et al., 2018; Steane et al., 2014). We discovered putative adaptive variation in wild mink through the identification of outliers in ATN1, IGF1 and neutral loci. In the context of our study, outlier alleles are likely domestic alleles that have been introduced to the wild population as a result of recent mink releases. The environmental variables used in the RDA support our hypothesis—we found latitude, longitude and road density are all significant influences on outlier alleles. Road density, which we used as a proxy for human population density and therefore also a high density of anthropogenic features, was only significant in neutral loci, and so, we think it unlikely that anthropogenic change is a factor in functional genetic diversity. However, as most of Ontario’s mink farms are in the southwestern section of the province (Figure 1), it is reasonable that the variables indicating spatial location are correlated to functional genetic diversity of wild mink populations. Since farms become less frequent the farther north or east one travels, the level of impact from a domestic release event should change as latitude and longitude changes, which is likely why we saw an association of latitude with IGF1 and longitude with AR and ATN1 (Table 5). Additionally, the sPCA of each functional marker shows a clear genetic distinction between individuals located in areas containing mink farms and those located far away from farms, namely those located farther north (Figure 5).

Interestingly, we found no evidence that mink farm density significantly affected functional allele frequency. In a past study, mink farm density was not the most informative variable in predicting a free‐ranging mink’s probability of being of domestic origin (Beauclerc et al., 2013). Due to a disparity in mink farm biosecurity measures, not all farms release individuals at the same rate, so some areas of high mink farm density have few escapees, while other areas of high mink farm density have a large number of escapees (Beauclerc et al., 2013). With this inconsistency, it is unlikely for RDA to identify mink farm density as a significant variable affecting functional allele frequency, even if the frequency differences are a result of domestic release events. Instead, in concordance with our study, Beauclerc et al. (2013) found latitude to be a better predictor variable.

A study of the native red fox populations suggested that rare haplotypes were the result of the release of captive fox from local fur‐farms which may have been replenishing their stock from native populations farther south (Mercon et al., 2017). Farmed mink in Canada have been domesticated for many generations, with relatively few wild mink used to supplement the domestic population due to disease concerns. There is also a commercial trade of domestic mink among farms. Overall, we consider the combined effects of the genetic founders in farms, inbreeding, drift and artificial selection to be the most likely processes leading to differentiation between farmed and wild mink in Ontario, as opposed to the introduction of wild mink alleles from distant locations. While functional genetic diversity of free‐ranging mink in areas near mink farms was not exceedingly different from those far away from farms, we advise genetic monitoring of American mink, because as domestic release events continue to occur, the disruption of genetic structure of wild populations surrounding farms will continue to accumulate. Ideally, protecting the genetic integrity of wild mink populations would require preventing domestic release events or establishing a protocol to mitigate the impact of these events. To decrease the number of these events in the future, we advise for increased fencing and security on farms, as recommended by the Mink Biosecurity Standard‐Producers Guide (Canadian Food Inspection Agency, 2016). If a release event does occur, a protocol for rapid response should be followed to reduce the probability of mink establishing feral populations. Countries such as Spain, Italy, and Estonia have recommended (a) installing surveillance systems to record presence of mink around farms, (b) preparing trapping and baiting equipment for immediate use and (c) training individuals to capture mink (Bonesi & Palazón, 2007).

## CONFLICT OF INTEREST

None declared.

## Data Availability

The data that support the findings of this study are openly available in Dryad at https://doi.org/10.5061/dryad.5mkkwh73g. (Morris et al., 2020)

## APPENDIX 1

**Table A1:** Primers, labels and annealing temperature (*T*_a_) used to amplify and genotype functional loci

Gene	Location	General function	Forward primer	Reverse primer	Label	*T*_a_ (°C)
ACTP	Exon	Growth	5′′‐ATG GGC GGG CCA GGG TTT TG‐3′	5′‐CAG AAA GGC CTC ATA GCG GTT CC‐3′	6‐Fam	52.4
AR	Exon	Behaviour	5′‐GCT GAC TGT TGT TGG GAA GGC‐3′	5′‐GCC ATC CAA GAC CTA TCG‐3′	Hex	61
ATN1	Exon	Stress	5′‐TCT TAG CCA ACA GCA ATG C‐3′	5′‐GAA TGG TGG GAG CTA CTG CTC T‐3′	Ned	55
DRD5	Exon	Behaviour	5′‐GCC CTT CCG CTA TGA ACG C‐3′	5′‐CGA AGA CCC CCA TGA TCA CCG‐3′	Hex	63.8
GRIN2B	Exon	Brain Development	5′‐CGG ATA TCT ACA AGG AGC‐3′	5′‐CGA ACG TGT CGT ACG AGT GC‐3′	Hex	56.4
HSPB7	Exon	Stress	5′‐CTC CTC CAC CTT CAG AGC‐3′	5′‐GCT GCC AAA ATC CTC G‐3′	6‐Fam	50.4
HTT	Exon	Behaviour	5′‐CCT CAA ATT GTA GAA ATG AAG GGC‐3′	5′‐GCC ACC ATC TTC AGA AGC‐3′	6‐Fam	63.8
IBSP	Exon	Growth	5′‐CAT TAC CAT TTT CTG CCT CTG TGC‐3′	5′‐GCC TTA GTA TAG CCT GAG TTG‐3′	Hex	61
IGF‐1	Promoter	Growth	5′‐GGG TAT TGC TAG CCA GCT GGT‐3′	5′‐CAT ATT TTT CTG CAT AAC TTG AAC CT‐3′	6‐Fam	54.2
NEUR0D1	Exon	Growth	5′‐CTC AGT TCT CAG GAC GAG GAG C‐3′	5′‐GCG CCT TAG CTT AAA ACG C‐3′	Ned	61
NFE2L1	Exon	Stress	5′‐CGA AGC CAT GCT GGA CGA GAT CAG C‐3′	5′‐GCA GAA CTT GGA GTA TTC GGG C‐3′	Hex	63.8
NR1D1	Exon	Clock	5′‐GTG TCA TCA CCT ACA TTG GC‐3′	5′‐GCT GGC AAT TTA CGC ACT GG‐3′	6‐Fam	63.8
PPP1R1B	Exon	Brain Development	5′‐GCT ATG AAC TGG GAG GGG TGC‐3′	5′‐GCA TTG CTG AGT CGC ACC TGC‐3′	Ned	63.8
RORC	Exon	Clock	5′‐TGT CAA GTT TGG CCG CAT GTC‐3′	5′‐TCA GGC AGG TCA GGT GAA GAG‐3′	Hex	61
TIMELESS	Exon	Clock	5′‐AGC AGG GGC CAG AGG AAC AAG‐3′	5′‐AGG CTC GCA CAA CAG TTG AGC‐3′	Ned	61
TNR	Exon	Growth	5′‐GCT TTC TGG GCA GCA GTT GC‐3′	5′‐CGG TGG TCC TGA AGA ACA TGC‐3′	Hex	63.8
TOB1	Exon	Growth	5′‐GAG AGG ACT GAG GTT TAG GGG GC‐3′	5′‐GAG AGG ACT GAG GTT TAG GGG GC‐3′	6‐Fam	61

**Table A2:** Allele frequencies for neutral microsatellite loci Mvi111, Mvi1272, Mvi1302, Mvi1016, Mvi114, Mvi4001, Mvi2243 and Mvi0072 in wild and domestic mink from three lines: Nova Scotia black (NSB), Ontario black (ONB) and Ontario brown (OND)

Locus	*F*_ST_	Allele	Allele frequency					Locus	*F*_ST_	Allele	Allele frequency				
			NSB	ONB	OND	Wild	Hybrid				NSB	ONB	OND	Wild	Hybrid
Mvi111	0.064	84	0.336	0.260	0.318	0.535	0.333	Mvi114	0.060	62	0.008	0.029	0.000	0.115	0.000
		88	0.000	0.010	0.000	0.004	0.000			68	0.371	0.337	0.227	0.216	0.333
		94	0.000	0.000	0.000	0.014	0.000			70	0.040	0.000	0.045	0.216	0.000
		96	0.022	0.130	0.045	0.106	0.167			72	0.000	0.000	0.000	0.011	0.167
		98	0.112	0.200	0.455	0.071	0.000			74	0.024	0.067	0.091	0.076	0.000
		100	0.358	0.230	0.136	0.071	0.333			76	0.331	0.385	0.591	0.115	0.167
		102	0.134	0.150	0.045	0.106	0.167			78	0.153	0.154	0.045	0.144	0.167
		104	0.000	0.000	0.000	0.025	0.000			80	0.065	0.029	0.000	0.065	0.000
		106	0.037	0.020	0.000	0.060	0.000			82	0.000	0.000	0.000	0.043	0.167
		108	0.000	0.000	0.000	0.007	0.000			84	0.008	0.000	0.000	0.000	0.000
Mvi1272	0.014	163	0.000	0.009	0.000	0.025	0.167	Mvi4001	0.179	223	0.632	0.651	0.545	0.086	0.333
		165	0.014	0.000	0.000	0.000	0.000			225	0.044	0.028	0.091	0.032	0.167
		167	0.049	0.009	0.125	0.105	0.000			227	0.235	0.264	0.273	0.795	0.500
		169	0.141	0.151	0.188	0.091	0.000			229	0.081	0.057	0.091	0.011	0.000
		171	0.141	0.170	0.188	0.076	0.167			231	0.000	0.000	0.000	0.036	0.000
		173	0.246	0.236	0.188	0.362	0.167			233	0.007	0.000	0.000	0.040	0.000
		175	0.338	0.302	0.313	0.264	0.500								
		177	0.035	0.057	0.000	0.040	0.000	Mvi2243	0.030	123	0.021	0.018	0.091	0.000	0.250
		179	0.035	0.066	0.000	0.007	0.000			127	0.000	0.000	0.000	0.004	0.000
		181	0.000	0.000	0.000	0.014	0.000			129	0.274	0.393	0.273	0.365	0.375
		183	0.000	0.000	0.000	0.014	0.000			131	0.000	0.000	0.000	0.022	0.000
										139	0.000	0.000	0.000	0.004	0.000
Mvi1302	0.055	207	0.198	0.177	0.182	0.018	0.000			141	0.000	0.000	0.000	0.007	0.000
		209	0.000	0.052	0.045	0.156	0.750			145	0.000	0.000	0.045	0.029	0.000
		211	0.009	0.000	0.000	0.018	0.000			147	0.021	0.027	0.182	0.113	0.125
		213	0.019	0.000	0.091	0.225	0.125			149	0.630	0.491	0.364	0.442	0.250
		215	0.255	0.135	0.182	0.283	0.000			151	0.000	0.000	0.045	0.000	0.000
		217	0.500	0.604	0.409	0.225	0.000			153	0.007	0.000	0.000	0.004	0.000
		219	0.000	0.010	0.091	0.043	0.125			155	0.048	0.071	0.000	0.004	0.000
		221	0.019	0.021	0.000	0.029	0.000			157	0.000	0.000	0.000	0.007	0.000
		223	0.000	0.000	0.000	0.004	0.000								
								Mvi072	0.078	257	0.000	0.000	0.000	0.007	0.000
Mvi1016	0.046	218	0.000	0.000	0.000	0.000	0.125			259	0.000	0.000	0.000	0.014	0.000
		220	0.000	0.000	0.000	0.050	0.000			261	0.787	0.714	0.455	0.430	0.875
		222	0.118	0.214	0.273	0.340	0.125			263	0.096	0.188	0.136	0.113	0.000
		224	0.007	0.036	0.045	0.124	0.000			265	0.037	0.000	0.091	0.261	0.000
		226	0.000	0.000	0.000	0.032	0.000			267	0.074	0.080	0.318	0.106	0.000
		228	0.201	0.152	0.045	0.099	0.125			269	0.007	0.018	0.000	0.067	0.125
		230	0.361	0.339	0.364	0.113	0.375			271	0.000	0.000	0.000	0.004	0.000
		232	0.056	0.107	0.091	0.113	0.125								
		234	0.236	0.152	0.000	0.128	0.125								
		236	0.021	0.000	0.182	0.000	0.000								
Number of individuals			76	56	11	144	4				76	56	11	144	4

**Table A3:** Allele frequencies for neutral microsatellite loci Mvi1321, Mvi099, Mvi1006, Mvi075, Mvi1354, Mvi1342 and Mvi002 in wild and domestic mink from three lines: Nova Scotia black (NSB), Ontario black (ONB) and Ontario brown (OND)

Locus	*F*_ST_	Allele	Allele frequency					Locus	*F*_ST_	Allele	Allele frequency				
			NSB	ONB	OND	Wild	Hybrid				NSB	ONB	OND	Wild	Hybrid
Mvi1321	0.021	90	0.000	0.009	0.045	0.035	0.000	Mvi075	0.034	105	0.000	0.000	0.000	0.003	0.000
		92	0.048	0.064	0.000	0.052	0.000			111	0.014	0.063	0.000	0.056	0.000
		94	0.123	0.109	0.227	0.269	0.125			113	0.000	0.000	0.000	0.003	0.000
		96	0.438	0.445	0.409	0.339	0.625			115	0.007	0.000	0.000	0.076	0.000
		98	0.027	0.055	0.045	0.147	0.125			117	0.336	0.384	0.364	0.122	0.333
		100	0.075	0.036	0.000	0.073	0.125			119	0.157	0.134	0.136	0.250	0.000
		102	0.027	0.082	0.091	0.031	0.000			121	0.057	0.036	0.136	0.083	0.000
		104	0.212	0.200	0.182	0.042	0.000			123	0.271	0.214	0.091	0.073	0.000
		106	0.000	0.000	0.000	0.004	0.000			125	0.043	0.116	0.136	0.038	0.333
		108	0.048	0.000	0.000	0.007	0.000			127	0.114	0.054	0.091	0.163	0.333
										129	0.000	0.000	0.045	0.080	0.000
Mvi099	0.065	320	0.000	0.000	0.000	0.004	0.000			131	0.000	0.000	0.000	0.045	0.000
		324	0.007	0.083	0.091	0.007	0.000			137	0.000	0.000	0.000	0.007	0.000
		326	0.014	0.000	0.000	0.011	0.000								
		328	0.000	0.102	0.182	0.000	0.125	Mvi1354	0.096	172	0.000	0.000	0.000	0.007	0.000
		330	0.079	0.056	0.227	0.000	0.250			176	0.000	0.000	0.000	0.209	0.000
		332	0.007	0.009	0.000	0.043	0.000			178	0.000	0.000	0.000	0.000	0.125
		334	0.000	0.000	0.000	0.004	0.000			180	0.000	0.000	0.000	0.022	0.000
		336	0.007	0.000	0.000	0.000	0.000			182	0.007	0.000	0.000	0.212	0.000
		338	0.236	0.287	0.091	0.193	0.375			184	0.051	0.009	0.000	0.047	0.125
		340	0.000	0.000	0.000	0.029	0.125			186	0.232	0.393	0.227	0.007	0.125
		342	0.086	0.093	0.091	0.386	0.125			188	0.014	0.000	0.000	0.032	0.250
		344	0.014	0.000	0.000	0.007	0.000			190	0.072	0.080	0.000	0.155	0.000
		346	0.086	0.074	0.182	0.018	0.000			192	0.000	0.000	0.091	0.115	0.000
		348	0.464	0.269	0.136	0.250	0.000			194	0.543	0.393	0.545	0.112	0.125
		350	0.000	0.000	0.000	0.004	0.000			196	0.080	0.107	0.136	0.079	0.250
		352	0.000	0.000	0.000	0.011	0.000			198	0.000	0.018	0.000	0.004	0.000
		354	0.000	0.009	0.000	0.021	0.000								
		356	0.000	0.000	0.000	0.007	0.000	Mvi1006	0.041	136	0.000	0.000	0.091	0.000	0.000
		358	0.000	0.019	0.000	0.000	0.000			144	0.000	0.000	0.000	0.004	0.000
		360	0.000	0.000	0.000	0.007	0.000			146	0.000	0.000	0.000	0.004	0.000
										150	0.324	0.264	0.182	0.077	0.500
Mvi1342	0.065	138	0.246	0.313	0.182	0.405	0.250			152	0.183	0.155	0.136	0.106	0.333
		140	0.000	0.000	0.000	0.004	0.000			154	0.303	0.418	0.545	0.278	0.000
		142	0.000	0.009	0.000	0.015	0.000			156	0.070	0.045	0.000	0.173	0.167
		144	0.200	0.143	0.318	0.113	0.250			158	0.021	0.009	0.000	0.099	0.000
		146	0.115	0.054	0.227	0.135	0.250			160	0.007	0.000	0.000	0.088	0.000
		148	0.000	0.036	0.091	0.000	0.000			162	0.007	0.018	0.045	0.092	0.000
		150	0.408	0.420	0.136	0.047	0.250			164	0.000	0.000	0.000	0.032	0.000
		152	0.008	0.027	0.000	0.077	0.000			166	0.021	0.082	0.000	0.039	0.000
		156	0.008	0.000	0.000	0.164	0.000			168	0.063	0.009	0.000	0.011	0.000
		158	0.000	0.000	0.000	0.037	0.000								
		160	0.000	0.000	0.000	0.004	0.000	Mvi002	0.034	180	0.000	0.000	0.000	0.004	0.000
		162	0.000	0.000	0.045	0.000	0.000			182	0.014	0.000	0.000	0.017	0.000
		164	0.008	0.000	0.000	0.000	0.000			184	0.000	0.000	0.000	0.042	0.125
		168	0.008	0.000	0.000	0.000	0.000			186	0.978	1.000	0.909	0.909	0.875
										188	0.007	0.000	0.091	0.028	0.000
Number of individuals			76	56	11	144	4				76	56	11	144	4

**Table A4:** Amplification success, annealing temperature and observed alleles at 38 candidate microsatellite loci

Gene	Clean amplification	*T*_a_ (°C)	Observed alleles (bp)				
			NSB	ONB	OND	Wild	Hybrid
ATN1	Yes	55	226, 232, 235	226, 235	232, 235	229, 232, 235	232, 235
AR	Yes	61	289, 295, 298, 301, 304	289, 298, 301, 304	289, 295, 298, 301	292, 295, 298, 301, 304	295, 298
IGF‐1	Yes	54.2	103, 105, 107, 111	103, 105, 107	103, 105, 107	97, 99, 101, 103, 105, 109, 111	103
TOB1	Yes	61	242, 245	242, 245	242, 245	242, 245	242, 245
ACTP	Yes	50	115	115	115	115	NA
DRD5	Yes	58.9	467	467	467	467	NA
GRIN2B	Yes	56.4	460	460	460	460	NA
HSPB7	Yes	50.4	156	156	156	156	NA
HTT	Yes	63.8	259	259	259	259	NA
IBSP	Yes	61	247	247	247	247	NA
NEUR0D1	Yes	62.7	229	229	229	229	NA
NFE2L1	Yes	62.8	343	343	343	343	NA
NR1D1	Yes	63.8	347	347	347	347	NA
PPP1R1B	Yes	63.8	271	271	271	271	NA
RORC	Yes	62.7	200	200	200	200	NA
TIMELESS	Yes	64.7	262	262	262	262	NA
TNR	Yes	63.8	465	465	465	465	NA
ADAMTS1	No	NA	NA	NA	NA	NA	NA
AKAP2	No	NA	NA	NA	NA	NA	NA
APBB1	No	NA	NA	NA	NA	NA	NA
ASCL1	No	NA	NA	NA	NA	NA	NA
CHERP	No	NA	NA	NA	NA	NA	NA
CLOCK	No	NA	NA	NA	NA	NA	NA
DYRK1A	No	NA	NA	NA	NA	NA	NA
HSP90AA1	No	NA	NA	NA	NA	NA	NA
LCORL	No	NA	NA	NA	NA	NA	NA
MECP2	No	NA	NA	NA	NA	NA	NA
MLL2	No	NA	NA	NA	NA	NA	NA
MTNR1B	No	NA	NA	NA	NA	NA	NA
OXTR	No	NA	NA	NA	NA	NA	NA
PAXIP1	No	NA	NA	NA	NA	NA	NA
PER1	No	NA	NA	NA	NA	NA	NA
PGC1B	No	NA	NA	NA	NA	NA	NA
PPPARGC1B	No	NA	NA	NA	NA	NA	NA
RXRB	No	NA	NA	NA	NA	NA	NA
SLC6A4	No	NA	NA	NA	NA	NA	NA
TRPC6	No	NA	NA	NA	NA	NA	NA
ZNF804A	No	NA	NA	NA	NA	NA	NA

**Table A5:** Percentage of overlap between pairs of population PCA ellipses

Locus	Population A	Population B				
		Wild	OND	ONB	NSB	Hybrid
AR (females)	Wild	—	6.46	3.34	5.66	0
	OND	82.88	—	19.99	40.37	0
	ONB	22.96	10.71	—	68.94	0
	NSB	41.7	23.21	74.02	—	0
	Hybrid	37.55	28.31	24.75	32.76	—
ATN1	Wild	—	0	0	43.09	40.49
	OND	100	—	100	100	100
	ONB	100	100	—	100	NA
	NSB	63.2	0	0	—	77.2
	Hybrid	75.2	0	0	95.32	—
IGF1	Wild	—	48.97	36.59	70.5	97.82
	OND	93.37	—	72.47	98.76	83.06
	ONB	91.15	94.7	—	93.29	92.54
	NSB	91.14	66.97	48.41	—	
	Hybrid	63.99	61.56	91.22		—
TOB1	Wild	—	0	53.07	25.03	0
	OND	96.92	—	33.89	0	0
	ONB	34.55	0	—	31.03	0
	NSB	19.39	0	36.93	—	0
	Hybrid	100	100	100	NA	—

Values represent the area (percentage) of Population A that is overlapped by Population B

**Table A6:** Microsatellite loci pairs under linkage disequilibrium

Population	Loci		*p*
NSB	ATN1	Mvi072	<.001
	Mvi099	Mvi072	<.001
ONB	Mvi111	Mvi114	<.01
Wild	Mvi111	Mvi1272	<.001
	Mvi1003	Mvi4001	<.001
	Mvi1302	Mvi1321	<.001
	Mvi111	Mvi1016	<.001
	Mvi4001	Mvi2243	<.001
	Mvi1272	Mvi075	<.001
	Mvi4001	Mvi075	<.001
	Mvi1006	Mvi1354	<.001
	Mvi1302	Mvi1354	<.001
	Mvi1006	Mvi072	<.001
	Mvi1302	Mvi1342	<.001
	Mvi1321	Mvi1342	<.001
